# Freestanding VO_2_ membranes on epidermal nanomesh for ultra-sensitive correlated breathable sensors

**DOI:** 10.1186/s40580-025-00476-3

**Published:** 2025-02-07

**Authors:** Dongha Kim, Dongju Lee, Jiseok Park, Jihoon Bae, Aiping Chen, Judith L. MacManus-Driscoll, Sungwon Lee, Shinbuhm Lee

**Affiliations:** 1 Department of Physics and Chemistry, Department of Emerging Materials Science, DGIST, Daegu, 42988 Republic of Korea; 2 Center for Integrated Nanotechnologies, Los Alamos National Laboratory, Los Alamos, NM 87545 USA; 3 Department of Materials Science and Metallurgy, University of Cambridge, 27 Charles Babbage Road, Cambridge, CB3 0FS UK

**Keywords:** Correlated breathable sensor, VO_2_, Freestanding membrane, Epidermal nanomesh, Sr_3_Al_2_O_6_, Tactile sensor, Respiratory sensor

## Abstract

The interest in highly sensitive sensors is rapidly increasing for detecting very tiny signals for Internet of Things devices. Here, we achieve ultra-sensitive correlated breathable sensors based on freestanding VO_2_ membranes. We fabricate the membranes by growing VO_2_ films onto sacrificial Sr_3_Al_2_O_6_ layer grown on SrTiO_3_, selectively dissolving the Sr_3_Al_2_O_6_ in water, and then rendering freestanding VO_2_ membrane on nanomesh. The nanomeshes are extremely flexible, sweat permeable, and readily skin-adhesive. The resistance of the VO_2_ membranes is reversibly tuned by human’s tiny mechanical stimuli and breath stimuli. The stimuli modulate the Peierls dimerization of one-dimensional V−V chains in the VO_2_ lattice which concomitantly controls the electron correlation and hence resistivity. Since our breathable sensors operate based on quantum-mechanical correlation effects, their sensitivity is 1−2 orders of magnitude higher than conventional tactile and respiratory sensors based on other materials. Thus, the freestanding membranes of correlated oxides on epidermal nanomeshes are multifunctional platforms for developing ultra-sensitive correlated breathable sensors.

**Graphical Abstract:**

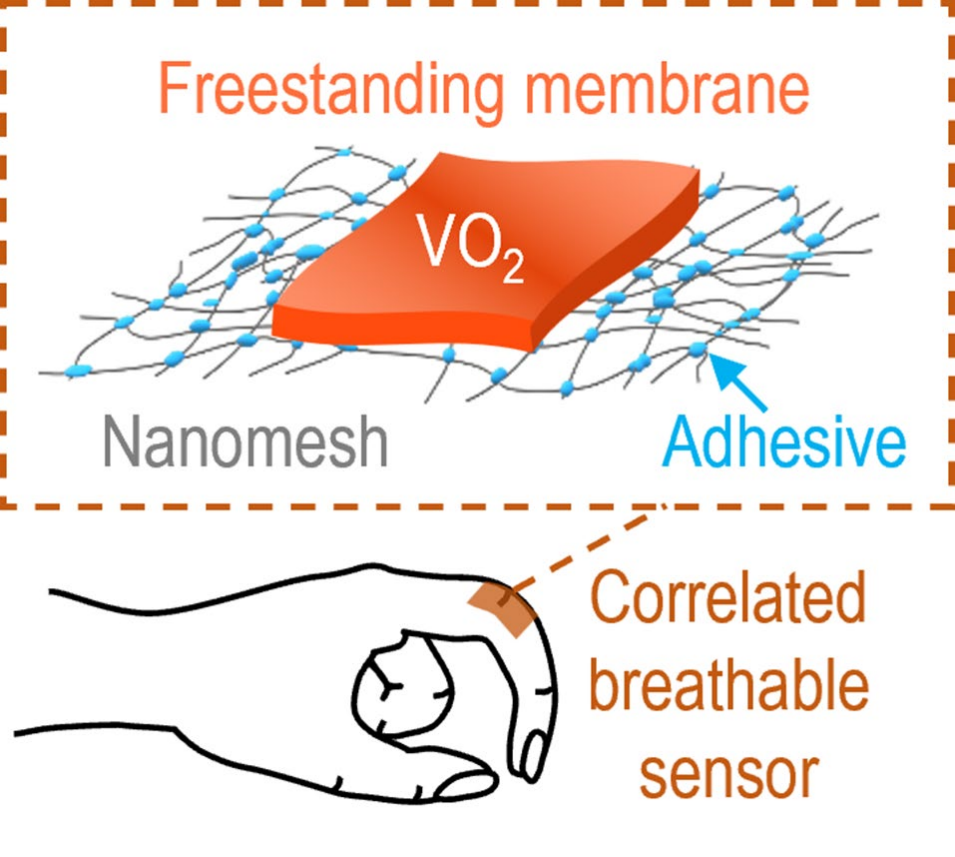

**Supplementary Information:**

The online version contains supplementary material available at 10.1186/s40580-025-00476-3.

## Introduction

Highly sensitive sensors are in high demand in Internet of Things era due to unprecedented sensitivity and spatial resolution even for very small signals. They are of particular interest for biomedical applications where tiny mechanical, chemical, and biological signals require sensing [1–4]. Recently, quantum sensing has become distinct and rapidly growing with spin qubits, trapped ions, and flux qubits that use quantum coherence, interference, and entanglement [5]. However, the current technology of quantum sensors uses materials which are large and heavy and not readily applicable to biomedical applications. For such applications, highly sensitive sensors, which are lightweight, breathable, flexible, comfortable to the wearer, and can operate at room temperature, are required.

Strongly correlated electron (SCE) oxides exhibit colossal changes of functionalities by external small stimuli since partially filled *d*- or *f*-electrons can be strongly correlated in narrow energy bands [6]. SCE oxides are quantum materials, with wide ranging physical properties, including metal-insulator transition, ferromagnetism, and superconductivity.

Nanometer-thick, flexible membranes can be created by removal from a water-soluble sacrificial buffer layer [7–16], or via remote epitaxy through graphene [17, 18]. Both methods enable the integration of high-quality membranes of quantum oxides on any substrate platforms. Bulk-like quantum properties were persistent in SCE membranes, including superconducting YBa_2_Cu_3_O_7−*x*_ [11], magnetoresistive La_0.7_Ca_0.3_MnO_3_ [13], ferrimagnetic Fe_3_O_4_ [14], and ferromagnetic SrRuO_3_ [16]. However, unsolved issues still remain. (1) Flexible oxide membranes are usually transferred onto non-breathable (non-sweat permeable) polymer substrates, such as polyethylene terephthalate (PET) and polyimide (PI). Thus, skin rashes are produced, and long-time usage of wearable devices is prohibited. (2) Both PET and PI are much less flexible than freestanding oxide membranes, and so the benefits of the flexibility of the oxide membranes are hindered, i.e. uniform coverage of skin wrinkles is prevented. (3) Non-adhesivity of PET and PI is detrimental to on-skin electronics.

In this work, we realized ultra-sensitive correlated breathable sensors by transferring SCE VO_2_ freestanding membranes onto epidermal nanomeshes after film removal from a water-soluble Sr_3_Al_2_O_6_ sacrificial layer. The nanomesh guaranteed sweat-permeability, extremely flexibility, and skin-adhesivity to the VO_2_ membranes. The VO_2_ membranes on the nanomesh had colossal resistance changes near room temperature which is a prerequisite for them to achieve sensitive resistance changes upon different stimulations. Indeed, the membranes showed 1−2 orders of magnitude resistance change even for tiny mechanical and breathing stimuli. The sensitivity is superior to conventional tactile and respiratory sensors.

## Correlated VO_2_ freestanding membranes on epidermal nanomesh

Among the different SCE oxides, VO_2_ was selected for this work owing to the presence of a colossal metal-insulator transition near room temperature [19–24]. Monoclinic structured VO_2_ (*a* = 5.74 Å, *b* = 4.52 Å, *c* = 5.38 Å, *β* = 122.6^o^) is an insulator at room temperature. In the presence of electric, thermal, optical, mechanical, and chemical stimuli, it goes through a first-order electronic transition into a metal with a concomitant structural transition into a tetragonal structured phase (*a* = *b* = 4.55 Å, *c* = 2.86 Å). Based on this versatility, historical examples of quantum phenomena in VO_2_ have demonstrated an orbital-occupancy-modified metal-insulator transition [25] and anomalously low electronic thermal conductivity [26]. However, until now, VO_2_ sensors have been fabricated as films deposited on solid substrates [27], membranes transferred onto flexible substrates such as PET and PI [28–30], and micro-/nano-structures dispersed on PET [31, 32].

Recently, we developed the epidermal nanomesh, which was breathable and adhesive on the skin [33, 34]. It offered excellent gas permeability with conformal integration to human skin. Owing to its great flexibility and permeability, it also provided great bio-compatibility as well as stretchability.

**Fig. 1:**
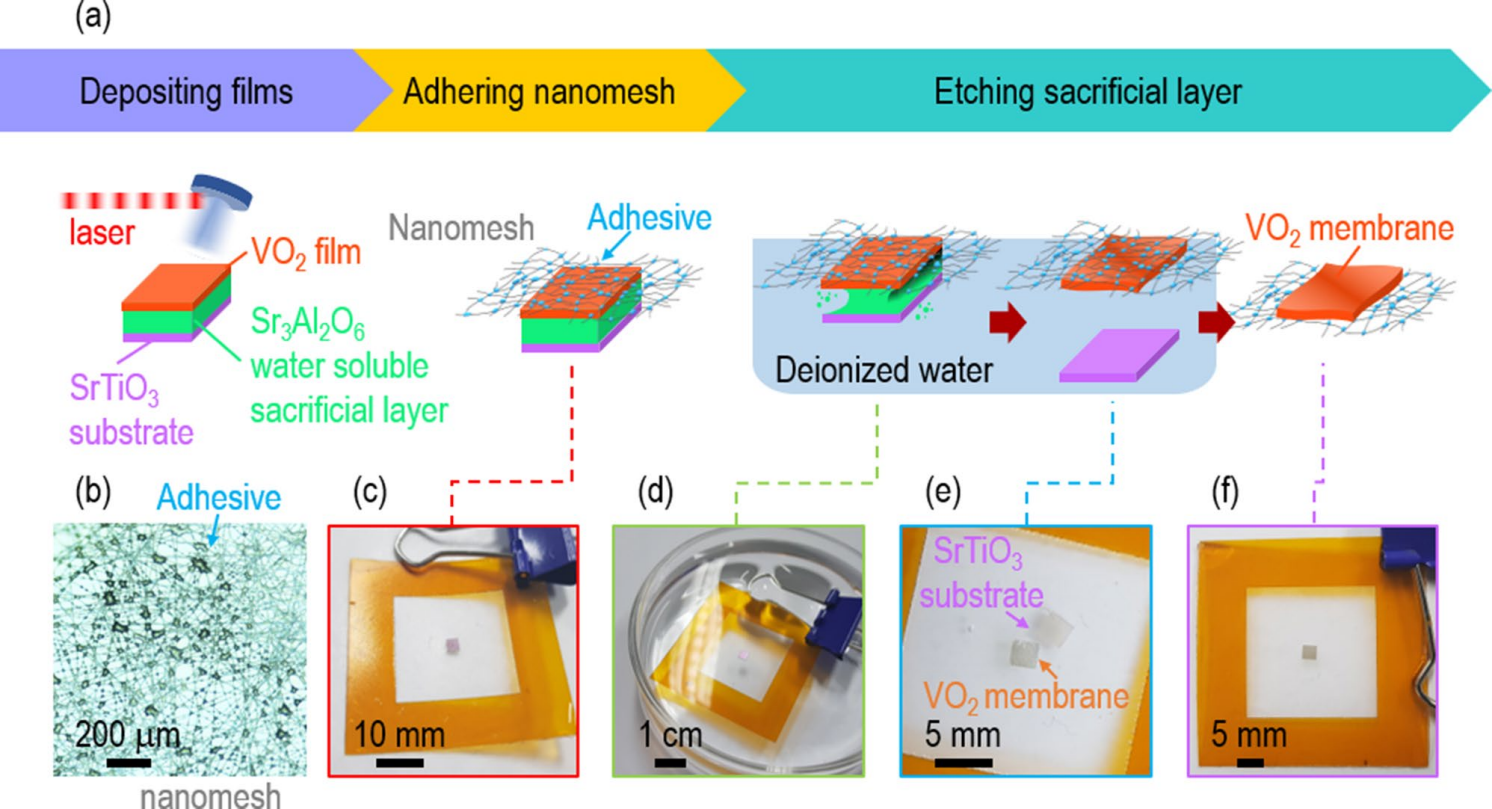
Novel process to transfer freestanding VO_2_ membranes onto epidermal nanomesh with assistance of water-soluble Sr_3_Al_2_O_6_ sacrificial layer. (**a**) There are three steps: depositing VO_2_/Sr_3_Al_2_O_6_ films on SrTiO_3_, adhering the VO_2_/Sr_3_Al_2_O_6_/SrTiO_3_ to nanomesh, and etching Sr_3_Al_2_O_6_ sacrificial layer. The photographs show (**b**) adhesives on nanomesh, (**c**) the VO_2_/Sr_3_Al_2_O_6_/SrTiO_3_ attached on the nanomesh, (**d**) selective etching of the Sr_3_Al_2_O_6_ sacrificial layer in deionized water, (**e**) VO_2_ membranes on the nanomesh with release of SrTiO_3_ substrate in deionized water, and (**f**) VO_2_/nanomesh

As illustrated by three steps in Fig. 1a, we fabricated correlated breathable sensors by transferring freestanding VO_2_ membranes on an epidermal nanomesh with the assistance of a water-soluble Sr_3_Al_2_O_6_ sacrificial layer. In the first step, we deposited Sr_3_Al_2_O_6_ films on (111)-oriented SrTiO_3_ substrates by ablating an Sr_3_Al_2_O_6_ pellet with a pulsed laser. We heated the substrates at 730^o^C under an oxygen partial pressure of 100 mTorr. Without breaking the vacuum, we in-situ deposited VO_2_ films on Sr_3_Al_2_O_6_-coated SrTiO_3_ substrates by ablating a V_2_O_5_ pellet with a pulsed laser under the deposition conditions of 450^o^C and 10 mTorr. In the second step, on the nanomeshes, which have uniformly distributed adhesives (Fig. 1b), we adhered the surface of the VO_2_ films (Fig. 1c). In the final step, we immersed a nanomesh/VO_2_/Sr_3_Al_2_O_6_/SrTiO_3_ sample in deionized water (Fig. 1d). Since Sr_3_Al_2_O_6_ is selectively dissolved by deionized water, we could separate SrTiO_3_ substrate from the VO_2_/nanomesh (Fig. 1e). It took ~ 6 h to fully dissolve the Sr_3_Al_2_O_6_ sacrificial layer and release the VO_2_ membranes from SrTiO_3_ (Figure S1). The size of the VO_2_/nanomesh was at least 2.5 × 2.5 mm^2^ and the thickness of the VO_2_ membranes was ~ 30 nm (Fig. 1f). We could stably reproduce more than 10 samples of the VO_2_/nanomesh. To compare the device performance of the VO_2_/nanomesh with that of conventional VO_2_/PET, we also transferred VO_2_ membranes on flexible PET (VO_2_/PET, Figure S2).

**Fig. 2:**
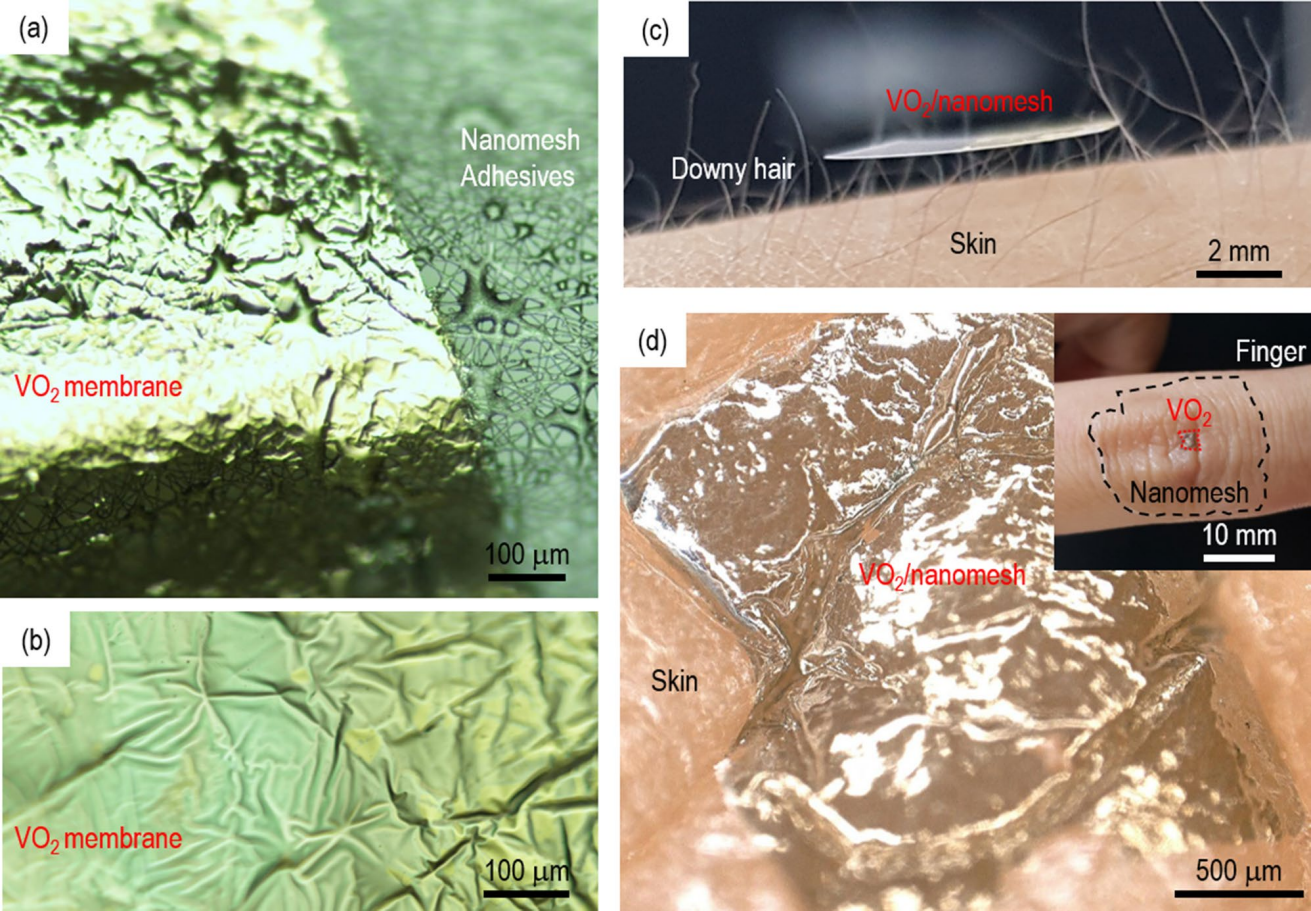
Advantages of VO_2_/nanomesh for correlated breathable sensors. The VO_2_ membranes (**a**) tightly sticked on nanomesh with help of adhesives; (**b**) are extremely flexible along the wrinkles of the nanomesh; (**c**) are lightweight and so can be floated on downy hair; (**d**) the adhesive nanomesh enables conformal integration of the VO_2_ membrane on the skin with gas permeability. The inset shows the VO_2_/nanomesh adhered on a finger knuckle

**Fig. 3:**
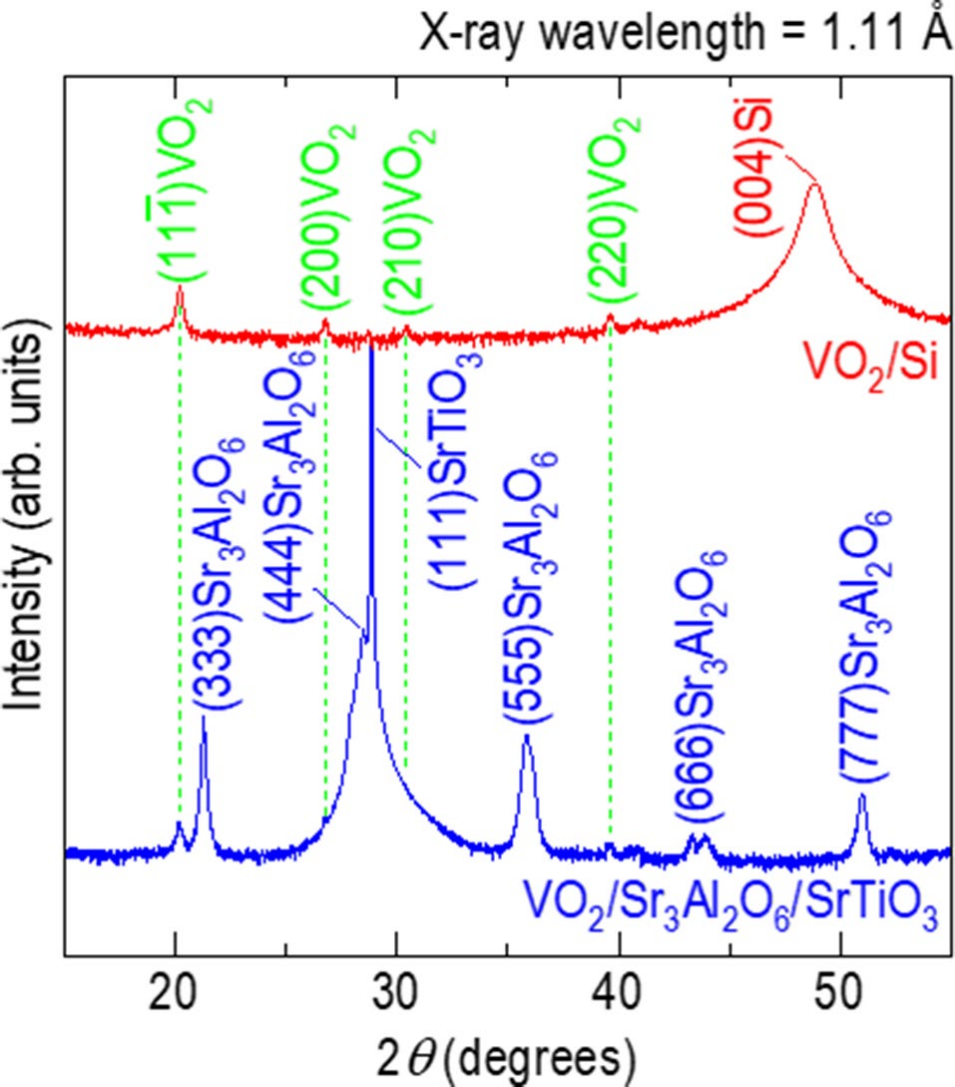
Monoclinic VO_2_ membranes. The peaks (green lines) in X-ray diffraction *θ*−2*θ* scans for both VO_2_/Si (red line) and VO_2_/Sr_3_Al_2_O_6_/SrTiO_3_ (blue line) correspond to those of monoclinic VO_2_ phase, which has been known to show colossal resistance change near room temperature. Additional peaks for VO_2_/Sr_3_Al_2_O_6_/SrTiO_3_ indicate the epitaxial growth of Sr_3_Al_2_O_6_ layer on (111)SrTiO_3_, which is available since the lattice parameters of cubic Sr_3_Al_2_O_6_ (*a* = *b* = *c* = 15.84 Å) were four times of those of perovskite cubic SrTiO_3_ (3.905 Å)

**Fig. 4:**
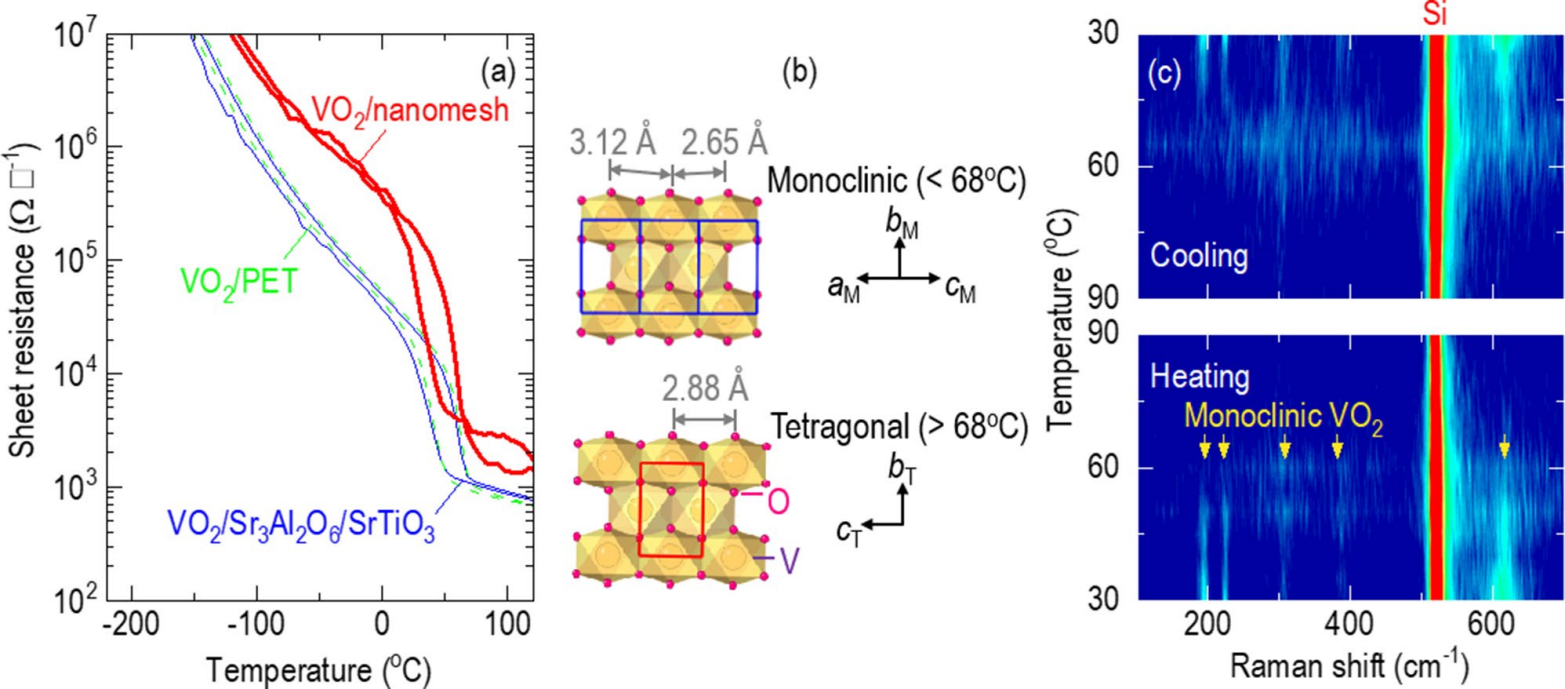
Reversible and colossal resistance changes of monoclinic VO_2_ membranes on nanomesh. (**a**) Our VO_2_/nanomesh (red line) shows two orders of magnitude resistance change with variation of temperature, which is larger than the one order of magnitude change in VO_2_/PET (green line) and VO_2_/Sr_3_Al_2_O_6_/SrTiO_3_ (blue line). All samples show a resistance change near 60.7 ± 1.1^o^C upon heating and near 37.3 ± 3.2^o^C upon cooling. (**b**) Low-temperature monoclinic VO_2_ (top) shows Peierls dimerization of one-dimensional V−V chains, different from the symmetric high-temperature tetragonal phase (bottom). The electron localization in V−V dimers results in a colossal resistance change between tetragonal and monoclinic VO_2_ structures. (**c**) Raman spectroscopy shows that the characteristic phonon modes of monoclinic VO_2_ (yellow arrows) disappear upon heating and reappear upon cooling at similar transition temperatures to the sheet resistance shown in (**a**)

**Fig. 5:**
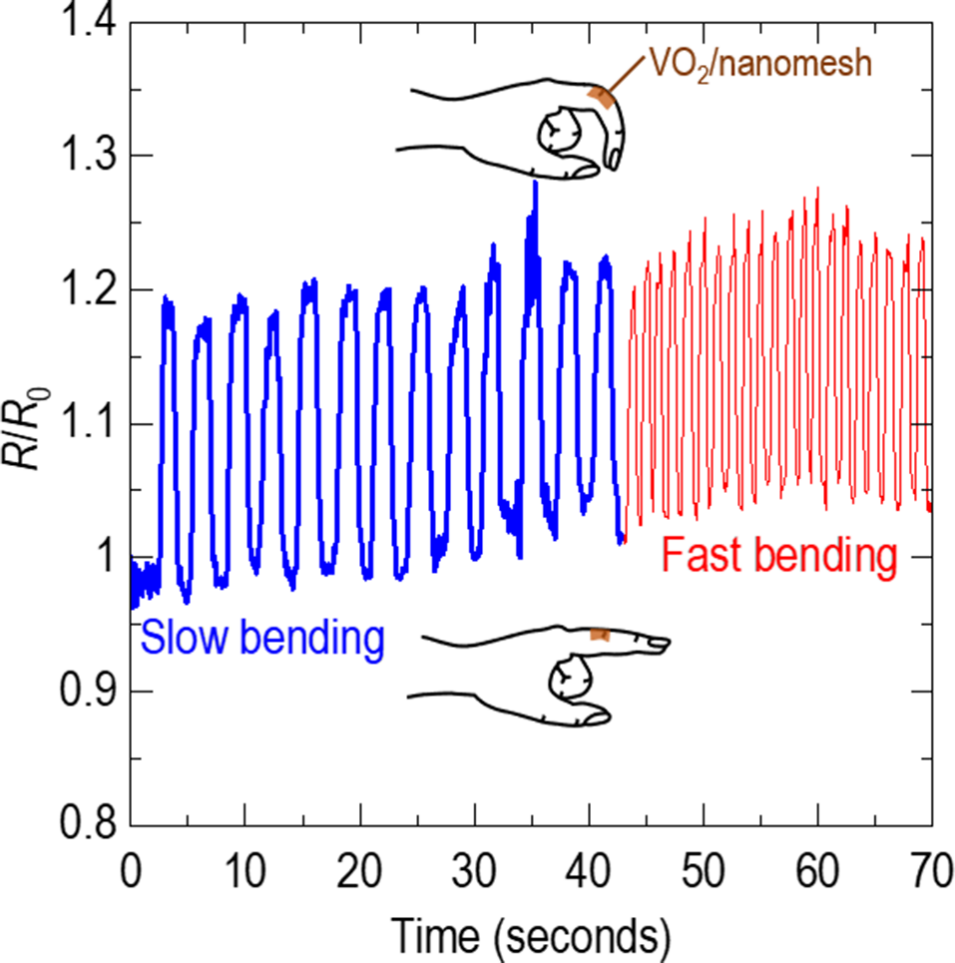
Highly sensitive tactile sensor of VO_2_/nanomesh. The resistance of the VO_2_/nanomesh, attached on the knuckle, increases when the finger is bent. When the finger is bent faster, the resistance of the VO_2_/nanomesh changes more quickly. Note that VO_2_/PET can not be tested as it is not be attached to the finger

**Fig. 6:**
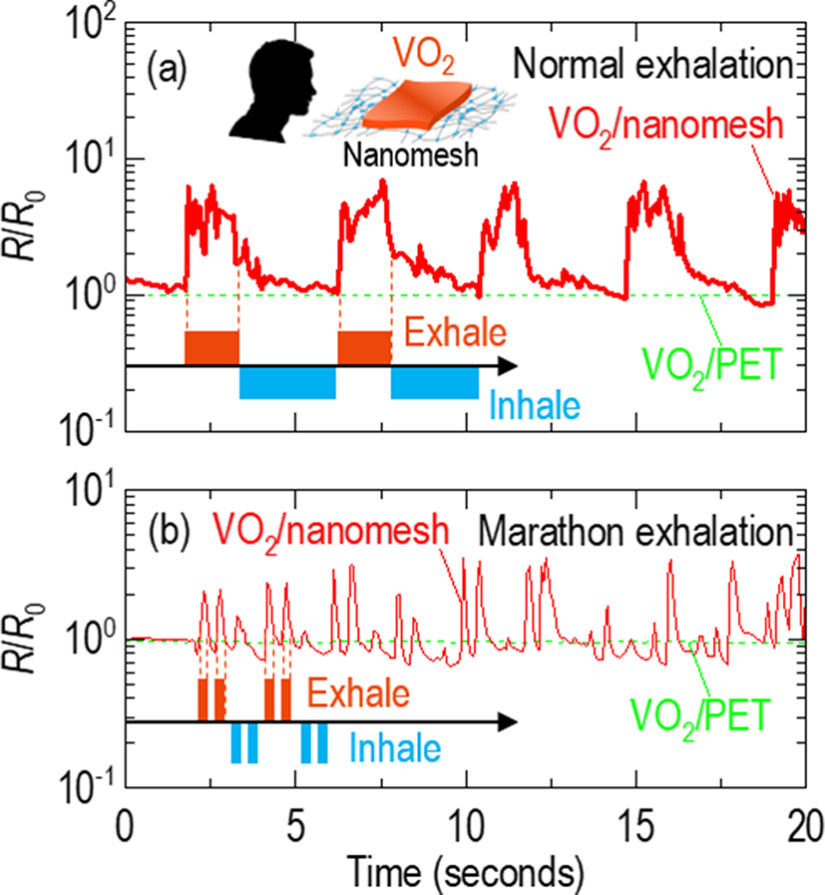
Highly sensitive respiration sensor demonstrated by VO_2_/nanomesh. The resistance of VO_2_/nanomesh increases when it is stretched with (**a**) ‘normal’ exhalation and (**b**) ‘marathon’ exhalation. The resistance change of VO_2_/PET is negligibly small in the respiration test (green line)

### Sweat-permeable, extremely flexible, and skin-adhesive VO_2_/nanomesh for correlated breathable sensors

The nanomesh made the transfer process of freestanding oxide membranes easier than conventional transfer methods. There were water droplets on the hydrophobic surface of PET and PI, and the oxide membranes on the water droplets tended to be slided and rolled [12, 27]. On the other hand, the deionized water was permeable through pores in nanomesh, suppressing the formation of water droplets and promoting selective etching of Sr_3_Al_2_O_6_. The side of oxide membrane which was interfaced with Sr_3_Al_2_O_6_ became a new surface. There was minimal dust on this clean surface since this side did not come into contact with organic supporters, e.g., polydimethylsiloxane, different from conventional transfer methods. Our transfer process was very safe, using only deionized water instead of dangerous acids [28]. It is also noteworthy that the expensive SrTiO_3_ substrates could be reused, as reported elsewhere [16].

The use of the adhesive nanomesh gave several advantages for the breathable quantum sensors over conventional sensors. With the help of adhesives, the 2.5 × 2.5-mm^2^-wide VO_2_ membrane was strongly adhered to the nanomesh (Fig. 2a), which allowed great flexibility of the membrane allowing it to stick along any wrinkles in the nanomesh without being crumbled, rolled, and torn (Fig. 2b). Since the VO_2_/nanomesh was lightweight, it could float on downy hair (Fig. 2c). The adhesives on both sides of nanomesh enabled the strong adherence of VO_2_ membranes on skin (Fig. 2d), so we could smoothly bend the VO_2_/nanomesh along with the finger motion (inset of Fig. 2d). The VO_2_/nanomesh was tightly adhered on the skin even when hands were washed. Since the nanomeshes have a polymer nanofiber-based porous structure, the conformal integration of VO_2_/nanomesh to the skin is possible with gas permeability.

### Persistent monoclinic structure of VO_2_ in membranes

The freestanding VO_2_ membranes had a monoclinic structure, which has been known to reversibly show colossal resistance changes near room temperature [19–24]. We investigated the crystal structure of freestanding VO_2_ membranes by X-ray diffraction (XRD) using an accelerator-based X-ray beam (> 11 keV, X-ray radiation with the wavelength of 1.11 Å). For this measurement, we transferred VO_2_ membranes onto Si substrates since the nanomesh and the PET substrates were easily burnt by very strong X-ray beam. The top of Fig. 3 shows an XRD *θ*−2*θ* scan of VO_2_/Si in the 2*θ* range of 15−55^o^ (red line). There are four tiny peaks at 20.2^o^, 26.7^o^, 30.4^o^, and 39.6^o^ (green lines. See Figure S3 for clarity.), corresponding to the four strongest peaks of monoclinic VO_2_. These polycrystalline XRD peaks of VO_2_ membranes also appeared in the VO_2_/Sr_3_Al_2_O_6_/SrTiO_3_ (blue line) system. The persistent XRD peaks of VO_2_ in both membranes (VO_2_/Si) and films (VO_2_/Sr_3_Al_2_O_6_/SrTiO_3_) indicated the non-destructive transfer of VO_2_ films from Sr_3_Al_2_O_6_-coated (111)SrTiO_3_ to the nanomesh as freestanding membranes.

### Reversible and colossal resistance changes of VO_2_/nanomesh near room temperature

The freestanding VO_2_ membranes showed reversible and colossal resistance changes near room temperature. The red line in Fig. 4a shows the temperature dependence of sheet resistance of the VO_2_/nanomesh in the temperature range between − 220 and 120^o^C. The sheet resistance of insulating VO_2_ membranes decreased upon heating, showing a large resistance drop by two orders of magnitude near 60.7 ± 1.1^o^C, which was recovered at a temperature of 37.3 ± 3.2^o^C upon cooling (Figure S4). The transition temperature of VO_2_/nanomesh was similar to that of VO_2_/PET and VO_2_/Sr_3_Al_2_O_6_/SrTiO_3_. This transition temperature, similar to ~ 68^o^C in the VO_2_ bulk [19–24], indicated that our VO_2_ membranes were strain free, while there is significant effect of substrate-induced strain on the transition of VO_2_ films [35]. Notably, the resistance changes in the VO_2_/nanomesh system was larger by one order of magnitude than the VO_2_/PET system (green line) and the VO_2_/Sr_3_Al_2_O_6_/SrTiO_3_ system (blue line).

The reversible resistance change in VO_2_ is accompanied by Peierls dimerization of one-dimensional V−V chains [36]. The V–V distance of 2.94 Å in VO_2_ is Goodenough’s critical V–V distance for the pairing of V^4+^ ions for metallic conduction. That is, one electron in the outermost V-3*d* orbitals are localized (free) when V–V distance is longer (shorter) than 2.94 Å, resulting in insulating (metallic) VO_2_. The electrical transition from insulating monoclinic VO_2_ to metallic tetragonal VO_2_ is accompanied by dissociation of V−V dimers, whose distances are 3.12 and 2.65 Å (top in Fig. 4b), to a uniform V−V distance of 2.88 Å (bottom in Fig. 4b).

A reversible change of the phonon mode, which we identified by Raman spectroscopy, indicated that our VO_2_ membranes underwent a temperature-driven Peierls dimerization of the V−V chains along with a monoclinic-tetragonal transition near room temperature. Figure 4c shows Raman spectra of VO_2_/Si in the wavelength range of 100–700 cm^−1^ upon the heating (bottom panel) and cooling (top panel) between room temperature and 90^o^C (See Figure S5 for raw data). We used VO_2_/Si since the Raman spectra of Si was dominantly isolated at a wavelength of 520 cm^−1^. At room temperature, the monoclinic VO_2_ showed complex phonon modes (yellow arrows), composed of stretching and bending of V−O−V and zigzag chains of V−V [22]. The suppression of the phonon modes above ~ 61^o^C upon heating indicated a transition into a more symmetric tetragonal VO_2_ in which the phonon modes dominantly include stretching modes of V−O−V [22]. The phonon modes of monoclinic VO_2_ were recovered below ~ 37^o^C upon cooling.

Similar transition temperatures of resistance changes (Fig. 4a) and phonon modes (Fig. 4c) indicated that structural and electrical transitions could be coupled to each other in our VO_2_ membranes. Therefore, we applied our VO_2_/nanomesh for the correlated breathable sensors using its resistance changes driven by the mechanical stimuli. As demonstrated in the next sections, our versatile VO_2_/nanomesh had superior sensitivity to conventional tactile and respiratory sensors, which were made with polymer nanofibers, metal nanowire, silk yarn, and MXene [37−43].

### Tactile sensor using VO_2_ membranes on nanomesh

Since the nanomesh is extremely flexible and adhesive, we attached the VO_2_/nanomesh on the finger knuckle (inset of Fig. 2d). Such a task was impossible with VO_2_/PET and VO_2_/Si. Figure 5 shows the reversible resistance changes of the VO_2_/nanomesh when we bent and straightened the fingers. When we bent the fingers, the resistance clearly increased by 20%. This increase of the resistance in VO_2_ membranes was consistent with localization of electrons in the elongated V−V dimer. When we straightened the fingers, the resistance was recovered to its original value. The faster we bent the finger, the faster the resistance changed, indicating that our VO_2_/nanomesh is an ultra-sensitive tactile sensor.

### Respiratory sensor using VO_2_ membranes on nanomesh

As shown by schematic in Fig. 6a, we breathed out over the VO_2_/nanomesh and simultaneously measured the resistance change as a function of time. Figure 6a shows the reversible resistance changes for a normal exhale. When we breathed out for 2–3 s (so-called ‘normal’ exhalation), the resistance of the VO_2_ membranes increased by one order of magnitude. Between the exhalation, the resistance was stably recovered to its initial value. The increase of the resistance in VO_2_ membranes was consistent with localization of electrons in the elongated V−V dimer. Figure 6b shows the resistance changes when we breathed out twice for a shorter time of 1 s (so-called ‘marathon’ exhalation). The VO_2_ membranes showed one order of magnitude increase of the resistance for each exhale. Notably, VO_2_/PET did not show any resistance changes in the respiratory test (green lines in Fig. 6 and Figure S6) since the PET was less flexible than the nanomesh. Using dry air with the constant temperature of ~ 18^o^C, which is far below the temperature (60.7 ± 1.1^o^C or 37.3 ± 3.2^o^C in Figure S4) for the metal-insulator transition, our VO_2_/nanomesh reliably sensed the resistance changes for 52 times (Figure S7), indicating negligible temperature effects. Therefore, our VO_2_/nanomesh is a sensitive respiratory sensor based on the mechanical stimuli.

## Conclusion

We fabricated ultra-sensitive correlated breathable sensors by transferring freestanding VO_2_ membranes on epidermal nanomeshes after removal of the membrane film from a water-soluble Sr_3_Al_2_O_6_ sacrificial layer. Our wearable devices were breathable, tightly stickable along the skin wrinkles, and reusable. These device properties cannot be achieved using polyethylene terephthalate and polyimide substrates. Due to the use of quantum-mechanical correlation effects, our VO_2_/nanomesh exhibited significantly larger reversible resistance changes by 1−2 orders of magnitude than conventional tactile and respiratory sensors. The combination of correlated oxide freestanding membranes and epidermal nanomeshes will stimuli great attention for developing multifunctional correlated breathable sensors.

## Experimental section

### *Deposition of VO*_*2 *_*film and Sr*_*3*_*Al*_*2*_*O*_*6 *_*sacrificial layer on SrTiO*_*3 *_*substrate*:

We used pulsed laser epitaxy to deposit a Sr_3_Al_2_O_6_ sacrificial layer and a VO_2_ film on a (111)-oriented SrTiO_3_ substrate. We ablated Sr_3_Al_2_O_6_ and V_2_O_5_ targets (Toshima Manufacturing Co., Ltd.), respectively, with Nd:YAG laser (Q-SMART 850; Quantel laser) with a wavelength of 266 nm, a laser intensity of 1 J cm^−2^, and a repetition rate of 10 Hz. The growth condition for the Sr_3_Al_2_O_6_ layer was a substrate temperature of 730^o^C and an oxygen partial pressure of 100 mTorr. It should be noted that SrTiO_3_ substrates have been widely used for growing a high-qulity Sr_3_Al_2_O_6_ sacrificial layer [7–16] since the lattice parameters of cubic Sr_3_Al_2_O_6_ (*a* = *b* = *c* = 15.84 Å) were four times of those of perovskite cubic SrTiO_3_ (3.905 Å). Therefore, we also used SrTiO_3_ substrates in this work. The Sr_3_Al_2_O_6_ sacrificial layer with flat surface would be helpful for minimizing the cracks in the VO_2_ freestanding membranes. We deposited the VO_2_ films at a substrate temperature of 450^o^C and an oxygen partial pressure of 10 mTorr. The metal-insulator transition in VO_2_ films generally disappeared when the films were thinner than ~ 20 nm [27]. Therefore, we transferred 30-nm-thick films to guarantee the transition in freestanding membranes.

### Fabrication of epidermal nanomesh:

We fabricate nanomesh substrates by electrospinning thermoplastic polyurethane (TPU). We disperse 13.5 wt% TPU elastomer solution (P22SRNAT, Miractran Co., Ltd) with mixture of methyl ethyl ketone (MEK) solvent and Dimethylformamide (DMF) in 5:5 weight ratio. The dispersed solution was stirred for 12 h at 80^o^C to sufficiently mix it. We filled a 10 mL syringe and placed it in the electrospinning system (ESR200R2, NanoNC). We applied 15 kV to perform electrospinning while ejecting a solution at 1.5 mL h^− 1^ to 2.5 × 2.5 cm^2^ polyimide frame. The ejected TPU elastomer filled up the polyimide frame within nanofiber-mesh structure. After that, we decorated a nanomesh with sticky polydimethylsiloxane (PDMS) by immersing the substrate into the PDMS and solvent mixture. We mixed the PDMS with hexane in 1:1 volumetric ratio to infiltrate PDMS on the nano mesh. After infiltration, we cured PDMS infiltrated nanomesh at 60^o^C for 6 h to remove hexane solvent.

### Characterization of VO_2_ membranes:

We investigated the structural properties using a six-circle high-resolution X-ray diffractometer (3A beamline; Pohang Accelerator Laboratory) that used the X-ray radiation with a wavelength of 1.11 Å. To measure the temperature dependence of resistance, we applied a current and measured the resistance (< 10 MΩ) upon heating and cooling using a physical property measurement system (Quantum Design, Inc.) in a four-point geometry with Pt pads. We recorded Raman spectra using a Raman microscope (inVia; Renishaw) in back scattering configuration. Each spectrum was the summed mean of seven individual spectra acquired at different locations on the sample through a 20× objective. We used the Raman laser with a wavelength of 532 nm and 50% power. For tactile and respiration tests, we measured the resistance of VO_2_ membranes using a source meter (2450; Keithley Instruments) in a two-point geometry with Pt pads.

## Electronic supplementary material

Below is the link to the electronic supplementary material.

Supplementary Material 1

## Data Availability

The data supporting the findings of this study are available from the corresponding author upon reasonable request.
