# Supplementary material for: Freestanding VO2 membranes on epidermal nanomesh for ultra-sensitive correlated breathable sensors
Source: Nano Converg. 2025 Feb 7;12:10. doi: 10.1186/s40580-025-00476-3 (PMC11806183; doi:10.1186/s40580-025-00476-3)
Supplement: Supplementary file 1 — Supplementary Material 1 [file 40580_2025_476_MOESM1_ESM.docx]

Supplementary Information

**Freestanding VO_2_ membranes on epidermal nanomesh for ultra-sensitive correlated breathable sensors**

Dongha Kim,^1,#^ Dongju Lee,^1,#^ Jiseok Park,^1^ Jihoon Bae,^1^ Aiping Chen,^2^ Judith L. MacManus-Driscoll,^3^ Sungwon Lee,^1,^* and Shinbuhm Lee^1,^*

^1^Department of Physics and Chemistry, Department of Emerging Materials Science, DGIST, Daegu 42988, Republic of Korea

^2^Center for Integrated Nanotechnologies, Los Alamos National Laboratory, Los Alamos, New Mexico, 87545 USA

^3^Department of Materials Science and Metallurgy, University of Cambridge, 27 Charles Babbage Road, Cambridge, CB3 0FS, UK

^#^D.K. and D.L. equally contributed to this work.

*E-mail: (Sungwon Lee) swlee@dgist.ac.kr

*E-mail: (Shinbuhm Lee) lee.shinbuhm@dgist.ac.kr

Keywords: Correlated breathable sensor, VO_2_, freestanding membrane, epidermal nanomesh, Sr_3_Al_2_O_6_, tactile sensor, respiratory sensor

Figures S1−S7

**1. Selective etching of Sr_3_Al_2_O_6_ layer in deionized water**


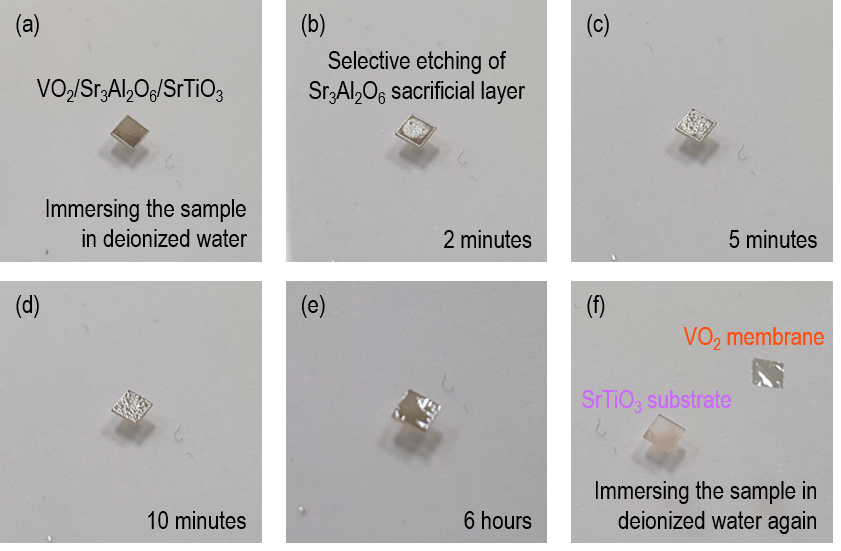


**Figure S1.** Snapshots for selective etching of Sr_3_Al_2_O_6_ layer in deionized water. (a) We immerse the VO_2_/Sr_3_Al_2_O_6_/SrTiO_3_ in deionized water. (b−e) The Sr_3_Al_2_O_6_ sacrificial layer starts to be dissolved within 2 minutes. We keep the sample in the deionized water for 6 hours to completely release the VO_2_ membranes from the SrTiO_3_ substrate. (f) When we immerse the sample in deionized water again, we can separate the VO_2_ membrane from the SrTiO_3_ substrate. We take the VO_2_ membranes from water and dry them for 60 minutes to remove water.

**2. Freestanding VO_2_ membranes on non-breathable polyethylene terephthalate**

**
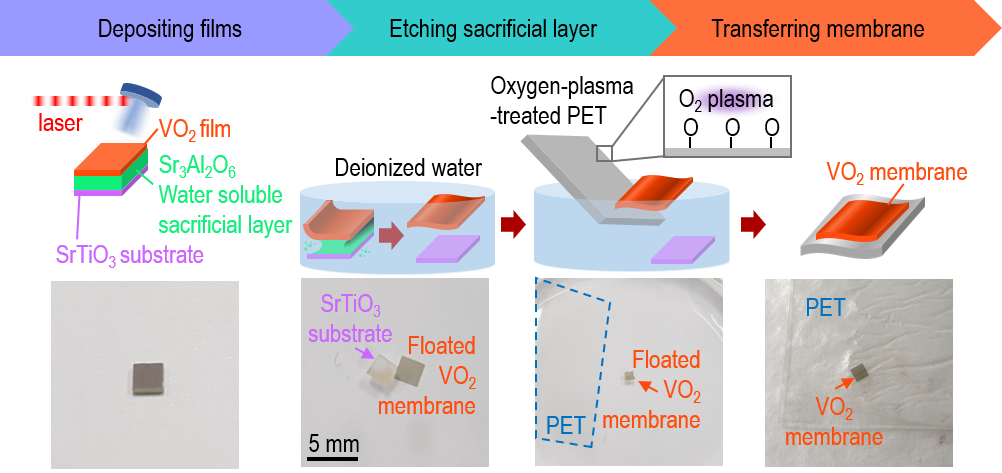
**

**Figure S2.** Conventional process to transfer freestanding VO_2_ membranes onto non-breathable polyethylene terephthalate (PET) with assistance of Sr_3_Al_2_O_6_ sacrificial layer. There are three steps: depositing VO_2_ films, etching Sr_3_Al_2_O_6_ sacrificial layer in deionized water, and transferring VO_2_ membrane on PET. The photos, from the left under the schematics, show VO_2_ films on Sr_3_Al_2_O_6_ sacrificial layer-coated SrTiO_3_ substrates, separation of VO_2_ membranes from SrTiO_3_ substrate after complete etching of Sr_3_Al_2_O_6_ sacrificial layer in deionized water, and scooping floated VO_2_ membranes by the oxygen-plasma-treated PET. The final photo displays the VO_2_/PET.

**3. Monoclinic VO_2_ phase in freestanding membranes**





**Figure S3.** Magnified X-ray diffraction of Figure 3, clearly showing monoclinic VO_2_ diffraction peaks (green-colored Miller indexes).

**4. Transition temperature for reversible and colossal resistance changes in VO_2_ upon heating and cooling**





**Figure S4.** Temperature dependence of $\frac{d(log(R))}{dT}$ (*R*: resistance of VO_2_, *T*: temperature). Independent of the substrates, all VO_2_ samples show negatively maximum $\frac{d(log(R))}{dT}$ values near 60.7 ± 1.1°C upon heating and near 37.3 ± 3.2^o^C upon cooling. Therefore, the temperature at which the resistance changes in VO_2_ membranes occur are similar to ~68^o^C of the VO_2_ bulk.

**5. Raw data of Raman spectra of VO_2_ freestanding membranes**


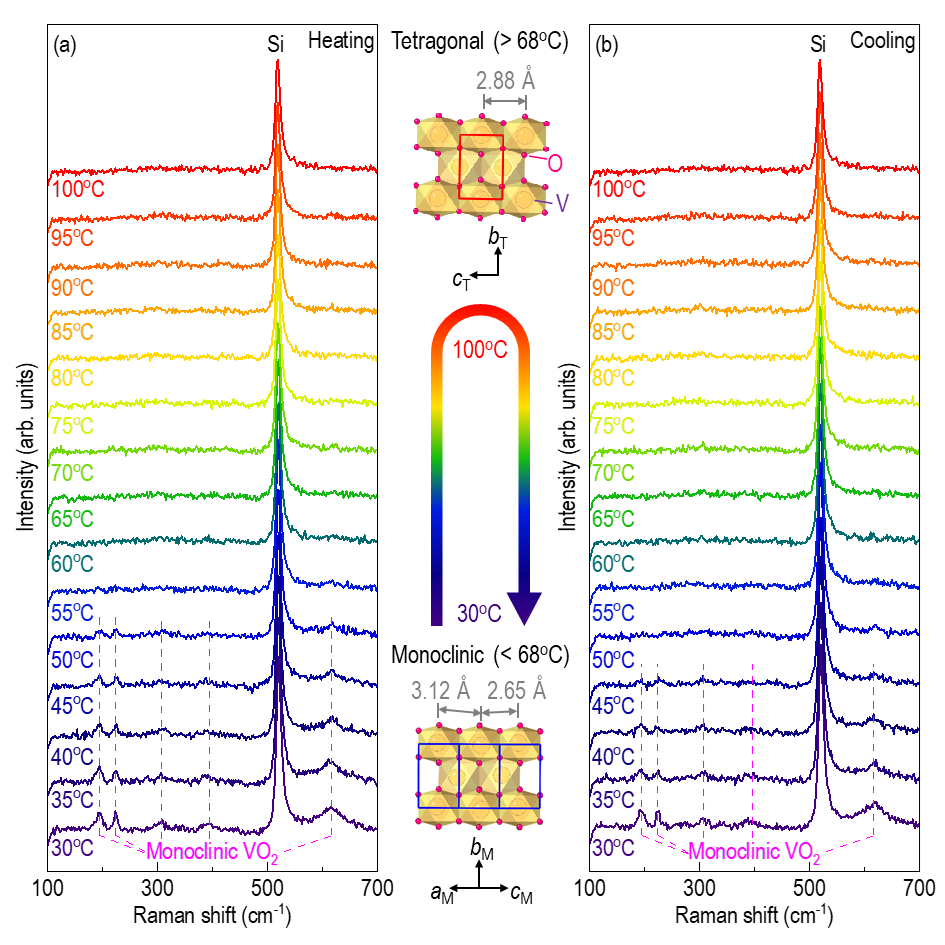


**Figure S5.** Temperature dependence of Raman spectra of VO_2_ freestanding membranes transferred on Si substrates. We acquire the spectra at each temperature between 30−100^o^C with 5^o^C interval upon (a) heating and (b) cooling.

**6. Poor performance of VO_2_/PET for respiration sensors**

**
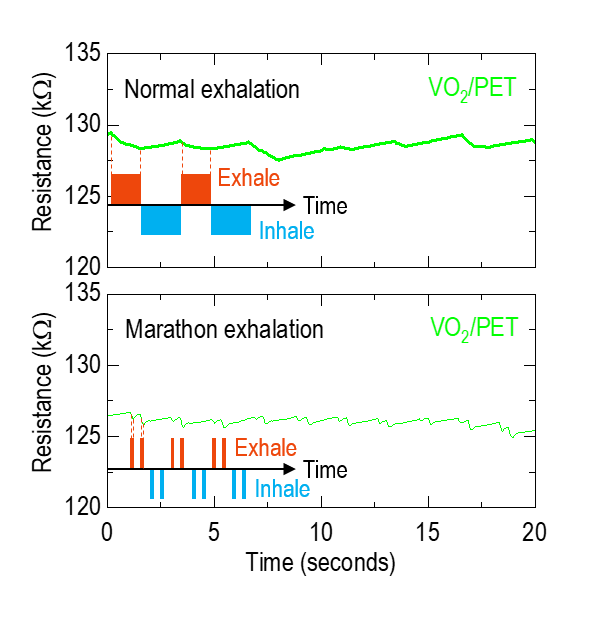
**

**Figure S6.** Respiratory test of VO_2_/PET. The resistance changes of VO_2_/PET are very weak with (a) ‘normal’ exhalation and (b) ‘marathon’ exhalation.

**7. Robust respiration sensors of VO_2_/nanomesh**

**
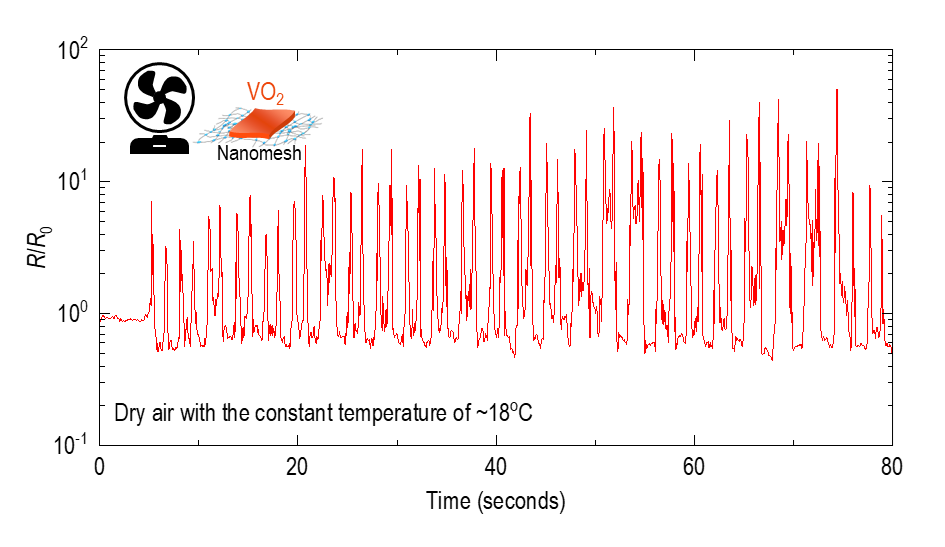
**

**Figure S7.** Long-term reversible resistance changes of VO_2_/nanomesh. When we blow dry air (with the constant temperature of ~18^o^C) towards the VO_2_/nanomesh, the breathable quantum sensor shows excellent endurance of resistance change up to at least 52 times. Between the exhalation, the resistance was stably recovered to its initial value.
